# Evaluating utility and feasibility of mismatch repair testing of colorectal cancer patients in a low-middle-income country

**DOI:** 10.1038/s41598-022-14644-6

**Published:** 2022-06-29

**Authors:** Inas Elsayed, Robert Geraghty, Salwa O. Mekki, Ahmed A. Mohamedani, Susan Ahern, Omer E. H. Salim, Balgis B. M. Khalil, Sawsan Abdelrahim, Suliman H. Suliman, Moawia M. A. Elhassan, Salah O. Salah, Mohamed E. Salih, Abubakr H. Widatalla, Osman S. Abdelhamed, Xiaosheng Wang, Éanna J. Ryan, Des Winter, Salih Bakhiet, Kieran Sheahan

**Affiliations:** 1 Biomedical Informatics Research Lab, School of Basic Medicine and Clinical Pharmacy, China Pharmaceutical University, Nanjing, China; 2 Cancer Genomics Research Center, School of Basic Medicine and Clinical Pharmacy, China Pharmaceutical University, Nanjing, China; 3 Big Data Research Institute, China Pharmaceutical University, Nanjing, 211198 China; 4 Department of Pharmacology, Faculty of Pharmacy, University of Gezira, P.O. Box: 20, Wad Madani, Sudan; 5 Department of Pathology, Centre for Colorectal Disease, St. Vincent’s University Hospital, Dublin 4, Ireland; 6 Department of Histopathology, Soba University Hospital, Khartoum, Sudan; 7 Department of Pathology, Faculty of Medicine, University of Gezira, P.O. Box: 20, Wad Madani, Sudan; 8 Department of Surgery, Soba University Hospital, Khartoum, Sudan; 9 Department of Histopathology, Ibn Sina Specialized Hospital, Khartoum, Sudan; 10 Department of Oncology, National Cancer Institute, University of Gezira, P.O. Box: 20, Wad Madani, Sudan; 11 Department of Oncology, Khartoum Oncology Hospital, Khartoum, Sudan; 12 Department of Surgery, Faculty of Medicine, University of Gezira, P.O. Box: 20, Wad Madani, Sudan; 13 Department of Surgery, Centre for Colorectal Disease, St. Vincent’s University Hospital, Dublin, Ireland; 14 Hull Royal Infirmary, Hull University Hospital NHS Trust, Hull, East Yorkshire UK; 15 School of Medicine and Medical Sciences, University College Dublin, Belfield, Dublin 4, Ireland

**Keywords:** Cancer, Genetics, Gastroenterology, Health care, Oncology

## Abstract

Molecular pathology services for colorectal cancer (CRC) in Sudan represent a significant unmet clinical need. In a retrospective cohort study involving 50 patients diagnosed with CRC at three major medical settings in Sudan, we aimed to outline the introduction of a molecular genetic service for CRC in Sudan, and to explore the CRC molecular features and their relationship to patient survival and clinicopathological characteristics. Mismatch repair (MMR) and BRAF (V600E) mutation status were determined by immunohistochemistry. A mismatch repair deficient (dMMR) subtype was demonstrated in 16% of cases, and a presumptive Lynch Syndrome (LS) diagnosis was made in up to 14% of patients. dMMR CRC in Sudan is characterized by younger age at diagnosis and a higher incidence of right-sided tumours. We report a high mortality in Sudanese CRC patients, which correlates with advanced disease stage, and MMR status. Routine MMR immunohistochemistry (with sequential BRAF mutation analysis) is a feasible CRC prognostic and predictive molecular biomarker, as well as a screening tool for LS in low-middle-income countries (LMICs).

## Introduction

Colorectal cancer (CRC) is a leading cause of morbidity and mortality worldwide^1,2^ Response to different therapeutic modalities varies due to tumour heterogeneity and patient characteristics. Advanced molecular techniques allow for classification of CRC into subtypes with distinct prognosis and response to anticancer therapies^3,4^.To date, the most robust CRC molecular classification is the consensus molecular subtype (CMS) one : CMS1 (microsatellite instability (MSI), immune), CMS2 (canonical/epithelial), CMS3 (metabolic/epithelial), and CMS4 (mesenchymal) subtypes^3^. However, in practice two main distinctions remain: MSI tumours which arise through the mismatch repair deficient (dMMR) pathway and mismatch repair proficient (pMMR)/microsatellite stable (MSS) tumours which develop through the chromosomal instability (CIN) pathway^5,6^. Sporadic dMMR tumours arise due to epigenetic silencing of the MLH1 gene, representing approximately 12% of all CRCs; a hereditary subset termed Lynch Syndrome (LS)) arise from constitutional mutations in the MMR genes (MLH1, MSH2, MSH6, PMS2) or the EpCAM gene upstream of MSH2, conservatively representing 2–3% of CRC cases^5,7^.

Precision medicine based upon molecular classification has revolutionised oncologic service provision. In recent years, medical treatment for patients with CRC has changed from “one size fits all” to a more tailored approach based on clinical characteristics and molecular profiling. Presently, the molecular changes with immediate implication for therapy are RAS (KRAS and NRAS), BRAF and MMR status^8^. Furthermore, new therapies directed against rare molecular alterations including HER-2 directed therapies and TRK-fusions are emerging^9^. However, to date the majority of research has been conducted in patients from high-income countries (HICs). Hence, molecular genomic services for CRC in LMICs represents a significant unmet clinical need and analysis of molecular markers such as MMR, and BRAF (either by molecular analysis or by mutation-specific immunohistochemistry) could improve the quality of health care provided in this setting^5,10^. In addition, MMR and BRAF analysis can provide insights into the likelihood of a LS diagnosis when performing screening for patients and their families and this can dramatically improve prognosis^5^.

There is a paucity of high-quality evidence on the molecular landscape of cancers found in low and middle-income countries (LMICs). While LMICs contend with barriers such as delays in accessing services, advanced malignancy at presentation, and restricted access to treatments, clinical research and practice in HICs is aimed toward developing personalised treatment strategies. As cancer medicine becomes increasingly driven by molecular changes in HICs, LMICs may be left behind. Realizing effective cancer molecular services is reliant on workforce training, equipment, infrastructure, and funding. Several approaches have been adopted in HICs to provide infrastructure and training to ensure that genomic information is incorporated into clinical practice. However, this is not the case in LMICs, where cancer molecular service development has been hampered by multiple challenges including lack of training of the medical workforce, and inadequate funding^11–13^.

The aim of this study was to firstly evaluate the molecular features of CRC and their relationship to clinicopathological characteristics in a cohort of Sudanese CRC patients. The secondary aim was to outline the challenges associated with implementing a molecular genetic service for CRC in Sudan.

## Materials and methods

### Study design

This was a joint project between the Centre for Colorectal Disease at St Vincent’s University Hospital, Dublin, Ireland & a number of hospitals in Sudan. The twin aims were a) to determine the feasibility of introducing universal MSI testing in CRC to a LMIC and b) to perform a retrospective cohort study to explore the clinicopathological features, MMR/BRAF status, and overall survival (OS) among Sudanese patients diagnosed with CRC at three major medical centres namely Soba University Hospital, Ibn-Sina Hospital, and Gezira Medical Laboratory in the time period from Jan. 1st 2016 to Dec. 31st 2018.

### Case selection and sample inclusion

#### Sample inclusion methodology

Pathological tumour material of all patients diagnosed with CRC at three major medical settings in Sudan from January 1st, 2016 to December 31st, 2018 were filtered. These included cases with biopsy or resection colorectal samples reported to fulfil criteria for CRC in the routine histopathology review. We identified a total number of 68, 60, & 61 cases diagnosed as CRC in Soba University Hospital, Gezira Medical Laboratory, and Ibn Sina Hospital in the specified period respectively. 50 patients were included based on the availability and quality of thematerial, specifically the overall appearance of the paraffin block, the amount of tumour and normal tissueavailable, and most importantly the quality of fixation and embedding of the tissue. After filtering, the selected cases included 29 cases from Soba University Hospital, 11 cases from Gezira Medical Laboratory, and 10 cases from Ibn Sina Hospital. 112 paraffin-embedded tissue blocks of CRC biopsies/resections from 50 CRC cases fulfilling the histopathological criteria of CRC were obtained. Blocks were selected to provide samples representing CRC and normal colon tissue for each case. Ultimately only samples with adenocarcinoma histopathological type (48 samples) that contains adequately processed primary tumor (44 out of the 48 samples) were processed for MMR expression and BRAF expression/mutation assessment.

### Demographic and clinical data

Data including age, gender, ethnic/sub-ethnic group, prior 10 years residence, history of malignancy, tumour anatomical site and clinical cancer stage (if available) were collected for all cases.

The diagnostic and therapeutic pathway for each patient is summarized (Table 1). Patient’s follow-up was conducted through review of medical records after follow up visits (Table 1) and personal contact (physical meetings or phone calls) if feasible. Data on neoadjuvant/adjuvant chemotherapy, CRC disease-free survival/recurrence (DFS), CRC recurrence, and CRC overall survival (OS) were collected if available. We evaluated CRC disease-free survival and recurrence if proven by either colonoscopy, imaging, or serum CEA elevation. We assessed CRC OS on confirmation of CRC related mortality when other causes of death were excluded.

**Table 1:** Protocol for diagnosis and treatment of CRC at the Sudanese Medical Centres.

Work-up of suspected CRC cases
1. Colonoscopy and biopsy,
2. CT scan (chest and abdomen)
3. CEA levels
4. Early stage CRC had surgery,
5. Advanced stages (Neoadjuvant therapy before surgery)
6. Adjuvant therapy was prescribed or not according to the post-operative stage
7. Patient follow-up after surgery : colonscopy (every 3 months for 1 year, then every 6 months for 2 years, and yearly for 10 years)

### Histopathological review

Histopathological characteristics were evaluated in the local hospitals; namely histopathological type, tumour grade, and tumour stage (Dukes and TNM stage) using standardised criteria^14^.

### Immunohistochemical analysis of MMR status and BRAF gene mutation expression

#### Immunohistochemistry

Immunohistochemistry **(**IHC) was performed at the Centre for Colorectal Disease, SVUH (RG). 112 blocks from 50 cases were examined. All paraffin blocks were cut on an automated Leica RM255 microtome and stained on a Leica ST5020 automated staining machine with an integrated Leica CV5030 glass coverslipper. All slides for immunohistochemical staining were baked in a 60 degree centigrade oven for two hours. H&E slides were microscopically re-examined by a Pathologist (SA) to confirm the presence of invasive CRC. 6 cases did not contain invasive carcinoma in any of the blocks received. No further testing was performed, and MMR IHC was performed on 44 cases.

#### Assessing MMR status

MMR status was assessed using IHC for MMR proteins, hMLH1 (BD Bioscience, clone G168-728), hPMS2 (BD Biosciences, clone A16-4, hMSH2 (Calbiochem, clone FE11) and hMSH6 (BD Biosciences, clone 44). Automated IHC was performed on the BOND instrument (Leica). The protocol involved heat-induced antigen retrieval with Bond Epitope Retrieval 2 solution for 30 min. Slides were incubated with antibody diluted 1 in 200 for 15 min at room temperature. Visualisation of the antibody antigen reaction was via the Bond Polymer Refine Detection kit (Leica).

Nuclear staining in any area of the tumour was classified as showing no loss of the mismatch repair proteins. Tumours showing complete loss of nuclear staining of the mismatch repair proteins in the entire tumour with concurrent positive staining of nuclei of non-neoplastic cells were classified as having loss of expression of that mismatch repair protein. Samples demonstrating MLH1 + PMS2 loss (*n* = 3) were examined by BRAF IHC and /or mutation testing if the staining was equivocal.

#### BRAF immunohistochemistry

Automated immunostaining was performed on the Ventana Ultra (Roche). The protocol involved dewaxing with Ezprep solution, followed by heat-induced epitope retrieval with CC1buffer for 64 min and then endogenous peroxidase inhibition. Slides were then incubated with the ready to use BRAF V600E mutation-specific monoclonal antibody, (Ventana, clone VE1 (CE-IVD)) for 16 min at 37 °C. Chromogenic detection was carried out using the OptiView DAB IHC kit (Roche) along with an Optiview amplification kit (Roche). Four minutes Optivew amplification H2O2 and four minutes Optiview amplification multimer incubation times were used. Slides were counterstained with haematoxylin II (Roche) for 4 min followed by bluing reagent (Roche) for 4 min.

Positive staining was seen as the presence of unequivocal cytoplasmic granular staining of any intensity in the tumour cells. Negative staining showed the absence of cytoplasmic staining in the tumour cells.

#### Molecular assessment of BRAF gene mutations

DNA was isolated from the tumour blocks using the Cobas DNA Sample Preparation kit (Roche). Real time PCR analysis of BRAF V600 mutation status with the BRAF/NRAS Mutation Test Roche Oncology Life Science Research (LSR) kit (Roche) was performed on the automated Cobas z480 analyser (Roche). A mutant control and negative control were included to confirm the validity of the analysis.

### Statistical analysis

All statistical analyses were performed using R (versions: 4.0.2 and 4.0.3) and STATA (version: 419.12.0.870). The Chi-square test was used to estimate correlation for categorical variables, while the Student t-test was applied for continuous variables. The Fisher exact test was used for small numbers where the conditions for the Chi-square test were not fulfilled. Kaplan–Meier survival analysis with log-rank test was used to estimate overall survival (OS) and its correlation with categorical variables while Cox regression was used for continuous variables. OS and DFS were calculated starting from the date of first diagnosis. Censor was death for OS and disease relapse and/or metastasis for DFS. All tests were two-tailed and a *P*-value of < 0.05 was considered statistically significant.

### Ethics declaration

This study was performed in line with the principles of the Declaration of Helsinki. Approval was granted by the Ethics Committees of Soba University Hospital, and National Cancer Institute, University of Gezira, Sudan.

#### Consent to participate

Informed consent was obtained from all individuals included in the study.

## Results

### Demographics

**Figure 1:**
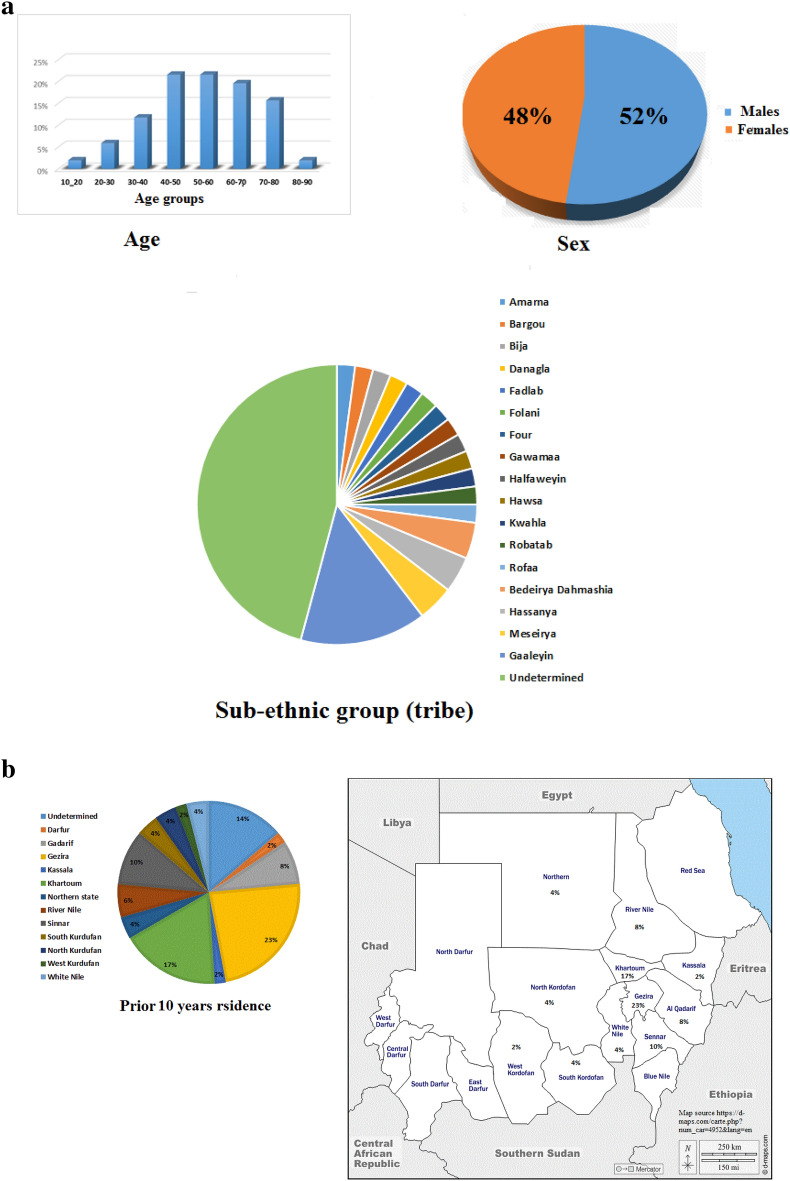
(**a**) Demographic data: Figure illustrates the age distribution, males and females percentages, and the sub-ethnic groups (tribes) of the studied CRC cases. (**b**) Prior 10 years residence of the studied cases: Figure illustrates the prior 10 years residence of the studied cases in Sudan’s 18 states. Sudan states map by D-maps.com, available from: https://d-maps.com/carte.php?num_car=4952&lang=en.

Cases included 26 males and 24 females, with age at diagnosis ranging from 13 to 85 years old and a mean age of 53 years (Fig. 1a). The mean age at diagnosis was similar in males and females (53). As patients self-determined their tribes we have identified several ethnicities including “Arabs”, “Nuba”, “Nubian”, “Fur”, “Hawsa”, and “Beja” (Fig. 1a). The prior 10 years residence of the cases were the central states of—“Gezira” and “Khartoum”- for the majority of cases, followed by “Sinnar” , “Gadarif”, “River Nile”, “Kurdofan”, “Northern ” ,”White Nile”, “Kassala” and “Darfur” states respectively (Fig. 1b).

### Histopathology

**Figure 2:**
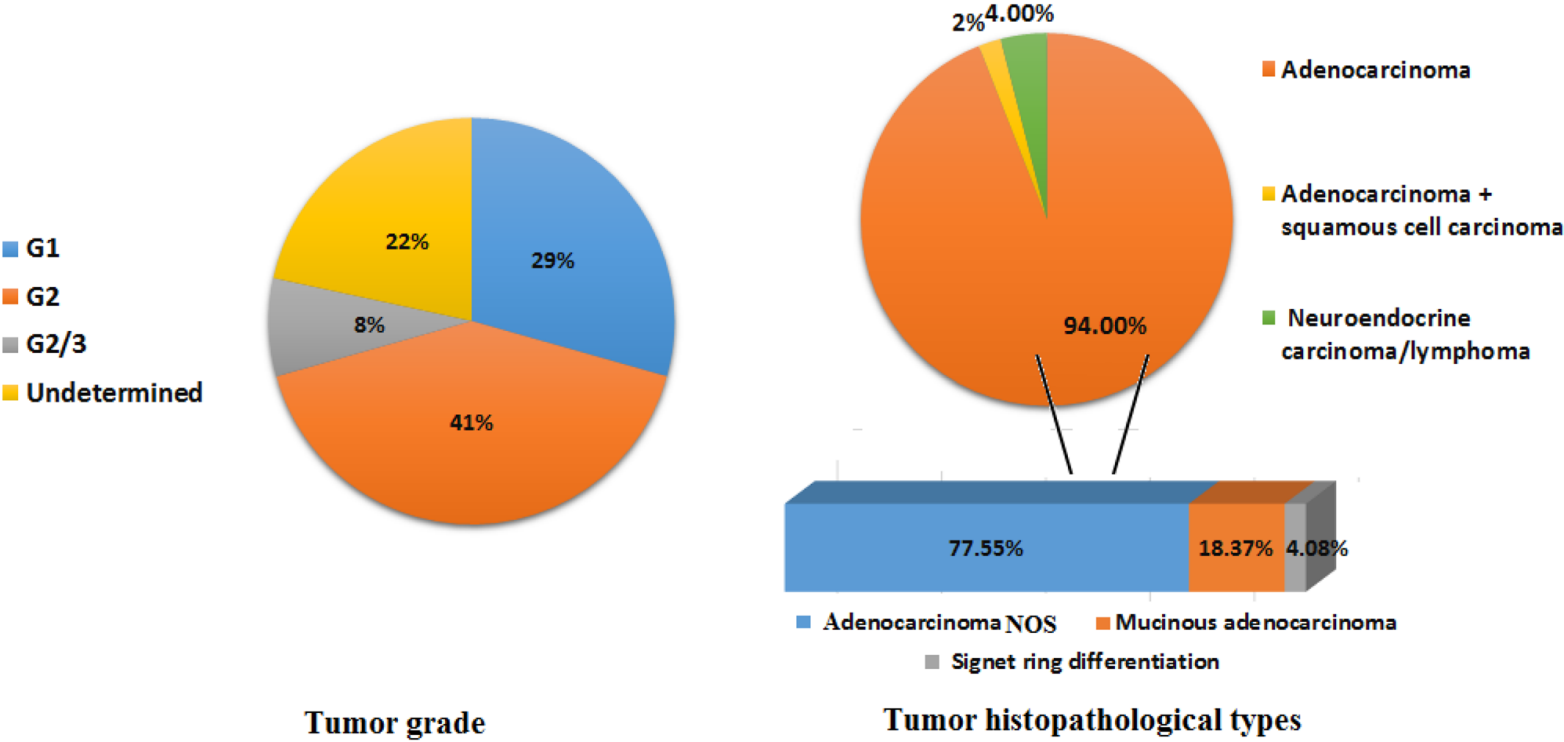
Histopathological characteristics: Figure summarizes the frequencies of: tumor differentiation grade, and tumor histopathological type.

94% of tumours were adenocarcinoma (78% adenocarcinoma NOS, 18% mucinous adenocarcinoma, and 4% signet ring adenocarcinoma), while 4% were neuroendocrine carcinomas, and 2% were combined adenocarcinoma and squamous cell carcinoma (Fig. 2). 2% were Stage I, 50% Stage II, 36% Stage III, 4% Stage IV, and 8% unstated stage. Metastasis was excluded in 32% of the cases, confirmed in 2% of cases and not assessed in 66% of the cases. 72% of the CRC tumours were low-grade tumours (G1, G2), 6% were high-grade tumours (G3) and 22% were ‘unstated grade’ (Fig. 2).

### Tumour anatomical location

In the left colon, the rectum was the most common site (36% with 18% involving recto-sigmoid colon), followed by descending colon (8%), sigmoid colon (4%) and splenic flexure (4%). In the right colon (16% of the cases), the most common site was the caecum (10%), followed by ascending colon (4%), transverse colon (4%) and right colon (specific location unspecified) (2%).We had the anatomic location of 8% of cases reported were in the colon (anatomic location unspecified) and cases were colorectal but location unspecified.

### MMR and BRAF status

**Figure 3:**
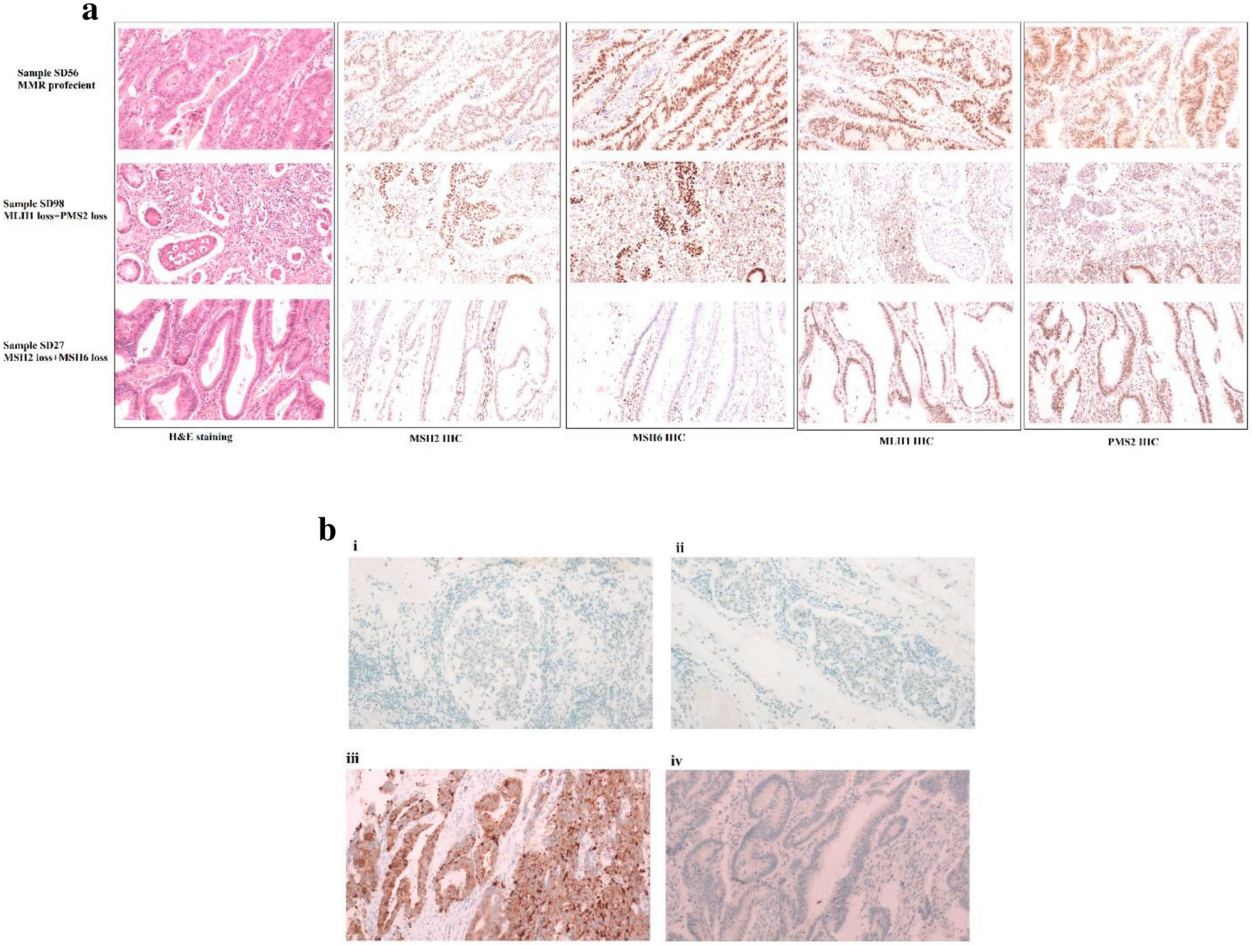
MMR and BRAF IHC : H&E and IHC profiles of (**a**) proficient MMR, dMMR (MLH1 + PMS2) and dMMR(MSH2 + MSH6) loss samples, (**b**) BRAF IHC of a dMMR (MLH1 + PMS2) sample where i. and ii. BRAF wild-type, iii. Positive BRAF control, iv. Negative BRAF control.

IHC analysis of the four MMR proteins expression (MLH1, MSH2, MSH6, and PMS2) revealed that 16% of the tumours were MMR deficient (dMMR). Of the dMMR samples; combined MLH1/PMS2deficiency constituted 43% (3 cases), and combined MSH6/ MSH2 deficiency represented 57% (4 cases) (Fig. 3). MMR deficiency was slightly more common in females (16.7% compared to 11.11%). In addition, using BRAF V600E IHC and/or BRAF molecular analysis, we found that the three tumours with dMMR (MLH1 & PMS2) were BRAF wild-type (Fig. 3). MLH1 promoter methylation status was not available in these cases and thus LS cannot be excluded except in one case > 60 years which is less likely to be LS. The cases (*n* = 4) with dMMR of MSH2 & MSH6 are presumed LS until proven otherwise, although it is conceivable that cases with double somatic mutations of MMR genes are also present in this subset. Table 2 compares the clinicopathological features of dMMR to proficient MMR CRC.

**Table 2:** Clinicopathological features of dMMR CRCs and pMMR CRCs.

	Deficient MMR	Proficient MMR	P value (Chi-square test/ student t-test)
Number of cases	7	37	
Age at diagnosis	Min 38	15	0.3 95% CI = (47.98132–56.5677)
	Mean 48.9	52.4	
	Median 48	55	
	Max 67	85	
	IQR 17	22	
Right-sided frequency	28.6%	10.8%	0.68
Rectal tumors frequency	43%	52.5%	0.68
Males	42.86%	56.8%	0.58
Females	57.14%	43.2%	0.58
Low tumor Grade	57.14%	75.7%	0.65
High tumor grade	0%	5.4%	0.7
Tumour stage	Stage I 0%	Stage I 3%	0.67
	Stage II 71%	Stage II 46%	
	Stage III 29%	Stage III 38%	
	Stage IV 0%	Stage IV 3% (10% stage unknown)	

* Chemotherapy protocol applied in The Radiation & Isotopes Centre Khartoum (RICK) & National Cancer Institute, Sudan:

Stages II/III:

1/mFOLFOX (modified FOLFOX) Folinic acid + 5FU + Oxaloplatin.

2/CAPETOX..Capecitabine + Oxaloplatin.

METASTATIC:

1/1/mFOLFOX + / − bevacizumab(Avastin).

2/mFOLFIRI ((modified FOLFOX) Folinic acid + 5Fu + Irinotican) + /  − bevacizumab(Avastin).

3/ CAPETOX + / − bevacizumab(Avastin).

### Survival of Sudanese CRC patients

**Figure 4:**
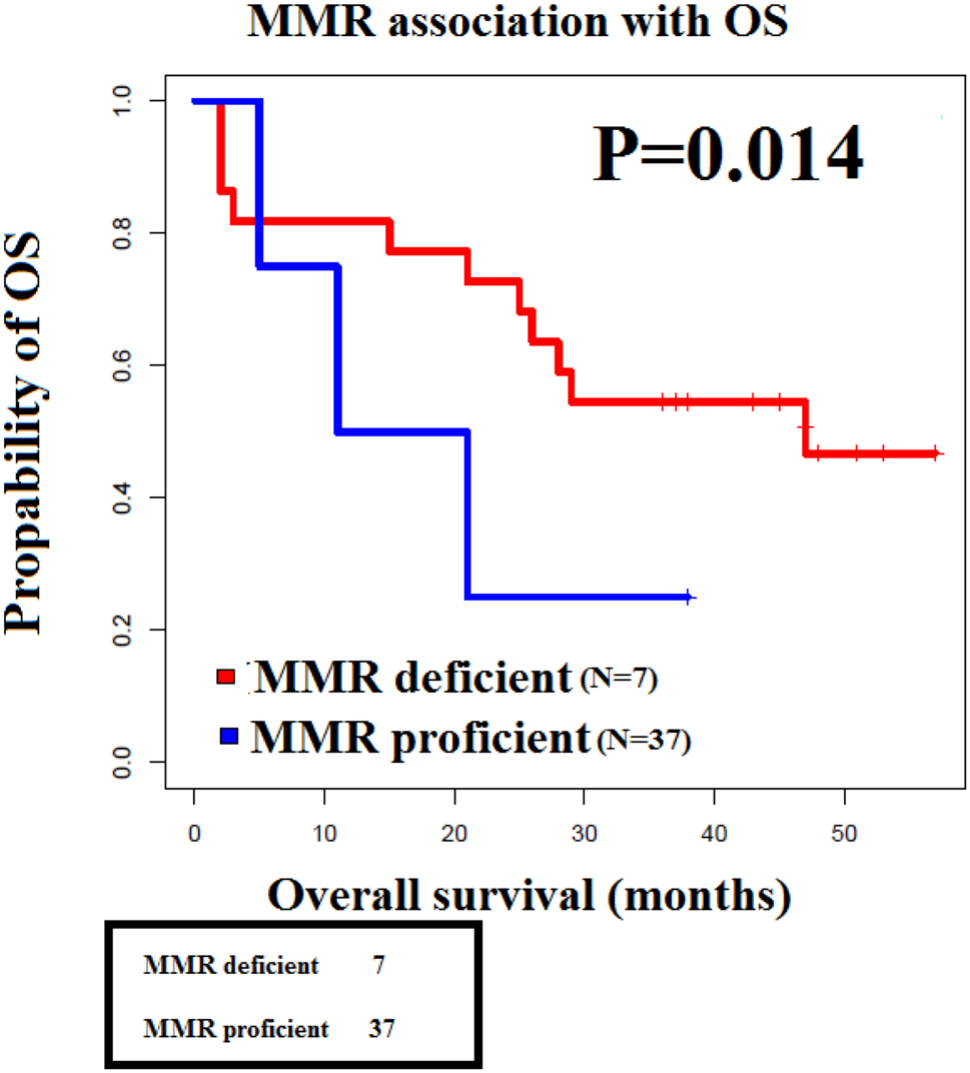
MMR status correlated with overall survival in Sudanese CRC patients: dMMR CRC showing worse OS compared to proficient MMR CRC.

16 of t50 cases (32%) died due to CRC within 5 years of diagnosis, 18 (36%) are alive, and 16 (32%) patients were lost to follow-up (Table 3). The mortality rate was highest in stage IV (50%), followed by stage III (44%), and lower in stage II (28%). We found that 3(43%) of the dMMR cases died within 5 years of diagnosis, 2 (29%) are alive and 2 (29%) were lost to follow-up (Table 3). The dMMR patient with the best OS (38 months) was stage T3N0M0 while the dMMR case with the worse OS (5 months) was stage T2N0MX. It is noteworthy that the M stage was not available in the records of 6 of the dMMR cases (Fig. 4).

**Table 3:** OS and DFS of dMMR CRCs compared to pMMR CRCs.

	dMMR (*n* = 7)	pMMR (*n* = 37)	*P* value
Mean Cancer-specific OS	Mean = 19 months	Mean = 32 months	0.014
OS	3 deceased (43%)	12 deceased (32%)	
	2 alive (29%)	14 alive (38%)	
	2 lost to follow-up (29%)	11 lost to follow-up (30%)	
1–5 years OS	< 1 year: *n* = 2 (~ 28.6%)	< 1 year: *n* = 4 (11%)	
	1–2 years: *n* = 1 (~ 14.2%)	1–2 years: *n* = 2 (5%)	
	2–3 years: *n* = 0 (0%)	2–3 years: *n* = 5 (13.5%)	
	3–4 years: *n* = 0 (0%)	3–4 years: *n* = 1 (3%)	
	> 5 years: *n* = 2 (~ 28.6%)	4–5 years: *n* = 9 (24%)	
	Missing: *n* = 2 (~ 28.6%)	> 5 years: *n* = 5 (13.5%)	
		Missing OS time: *n* = 11 (30%)	
5 years DFS	5 years DFS: *n* = 2 (28%)	5 years DFS: *n* = 9 (24%)	0.35

With regard to disease-free survival (DFS) we observed that 10% of patients developed recurrence, 4% had disease progression (distant metastasis), and 2% developed extra-colonic cancers in less than 5 years.

## Discussion

In this study, we have demonstrated the need for introducing cancer molecular services for CRC in Sudan. This was performed by analyzing the MMR and BRAF mutational status, clinicopathological and survival patterns in a cohort of patients with CRC attending three major Gastroenterology settings in Sudan. We applied IHC analysis of MMR and BRAF-analysis, as an algorithm for MSI assessment for LS and followed the patients for 5 years to report disease survival^5,15^.

There is emerging evidence that the molecular landscape of cancer differs geographically and by genetic ancestry, which cannot be explained by environmental factors. Several reports indicate a higher incidence of dMMR CRC among African patients (including native Africans and African Americans)^16–18^. We have demonstrated a dMMR CRC frequency of 16% in this cohort, which is in contrast to the only previous report of dMMR CRC frequency in Sudan by Zakout et al. of a 9.5% dMMR CRC rate. The variation in the reported frequency indicates the need for wider screening to accurately estimate the dMMR CRC frequency in Sudan^19^.

CRC presentations in several African countries- including Sudan- are characterized by younger age and rapid progress of the disease^20^. Numerous health and socioeconomic factors are implicated in the development of early-onset CRC (EORC) in Africa including a possible higher prevalence of LS among CRC African patients; however, additional studies are needed to validate such claims^20^. Similarly, EOCRC cases are reported in Sudan although it is hard to specifically describe the disease patterns based on the limited published reports^21,22^. The higher frequency of rectal tumours (43%) combined with the young age of the dMMR cases of our cohort may suggest either EOCRC or a higher LS incidence^23,24^. It is important to note that the possibility of LS cannot be ruled out in dMMR CRC regardless of family history, especially in those with young age CRC onset (57% of the cases were underthe age of 50) and cases with loss of MLH1 and PMS2 which have a wild-type BRAF status^25^. Unfortunately, analysis of MMR germline mutation and MLH1 methylation status was not possible to stratify these cases further highlighting the need for the development of cancer genomics in LMICs. Presence of other Lynch-like hereditary syndromes is another possibility that would require similar advanced molecular genetic services to elucidate^26^.

CRC in our cohort was found to have a higher mortality than that reported for CRC in Western populations, and dMMR CRC cases, specifically, had a worse survival in this cohort compared to pMMR. It is difficult to draw conclusions about dMMR cases OS in Sudan considering the limited sample size of our study, however, the higher frequency of rectal tumors in the Sudanese dMMR CRC patients (43%) may explain the observed worse OS. Unlike MSI-H colon cancers, MSI-H tumors are associated with a worse survival in rectal cancers. This could be due to a higher frequency of Lynch syndrome among MSI-H rectal tumors, or could be a consequence of the poor response of MSI-H rectal cancer to the 5-FU-based neoadjuvant and/or radiotherapy^23,24,27^. Another potential explanation could be under-staging of the dMMR cases with missing M stage data, which is plausible considering the inefficiency in communicating patient’s data between different hospitals and hospitals’ units in Sudan due to lack of automated clinical information systems. Generally, these findings highlight the need for molecular testing of CRC in Sudan and other LMICs to allow for CRC prognostic stratification; as a predictive marker of response to chemotherapy and novel immunomodulatory agents; and as part of the algorithm for diagnosing LS. Currently, no test for CRC molecular subtyping is routinely applied for Sudanese patients, and diagnosis of CRC at the three medical settings is based on clinical presentation, colonoscopy & tumor histopathology, CEA levels, and CT scanning. During this study, a universal protocol and standard operating procedures were developed in collaboration with the Centre for Colorectal Disease in an attempt to standarise pathological diagnosis and treatment of all CRC based on tumour type (colon or rectal) taking account of the patient’s MMR and BRAF status (supplementary document). Evaluation of MMR status of CRC patients may identify MSI CRC and suspected LS, or other hereditary syndromes cases and assist in tailoring the therapeutic protocols to improve the prognosis and clinical outcomes of these CRC types in Sudan^5,28^. Introducing such services to countries with limited resources appears feasible through applying lower-cost tests, such as IHC, with universal MSI/MMR testing particularly given the young age profile of CRC patients in Sudan^26^. Moreover, the availability of newer technologies (e.g., the Idylla MSI and BRAF assays), which demonstrates comparable sensitivity and specificity to IHC and molecular MSI assays may provide efficient, less laborious and cost-effective options suitable for LMICs health systems^29,30^. Considering the high CRC mortality reported in our study, applying precision medicine is anticipated to reduce the observed mortality in Sudanese medical settings, however, many challenges willremain. The lack of infrastructure, finance, logistics, and trained laboratory personnel are major challenges facing precision medicine in Sudan similar to other developing countries^11,12,31–33^. Furthermore, low socio-economic status, limited access to medical and surgical services, and socio-cultural barriers are additional obstacles^34,35^. In addition, the presence of the majority of Gastroenterology services in the central states of Sudan compared to distant states, limits the accessibility of precision medicine services. This is clearly demonstrated in our study (Fig. 1b), where remote states are less represented in the number of diagnosed cases.All these challenges need to be addressed to successfully implement precision medicine treatment.

In conclusion, our study shows that dMMR/MSI CRC represents 16% of the CRC Sudanese cases, and 86% of the dMMR cases were suspected LS. The OS of CRC in our cohort was poor indicating a need for optimizing therapeutic protocols. We recommend additional research with larger sample sizes to investigate the exact frequency of dMMR in Sudan and to evaluate risk factors, clinicopathological features and clinical outcomes. We also recommend universal MMR IHC testing as a routine for all CRC patients in Sudan to screen for LS, inform prognosis, and direct medical & surgical therapy. Performing MMR IHC on biopsies has been proven to be as beneficial as on surgical resections with many advantages including better fixation^36^. In the long term this is where LMI countries should be moving once endoscopic services improve.

## Supplementary Information

Supplementary Information.

## Data Availability

Raw data of this study cohort is available upon reasonable request from the corresponding author.
