# Supplementary material for: Evaluating utility and feasibility of mismatch repair testing of colorectal cancer patients in a low-middle-income country
Source: Sci Rep. 2022 Jun 29;12:10998. doi: 10.1038/s41598-022-14644-6 (PMC9243080; doi:10.1038/s41598-022-14644-6)

## Evaluating utility and feasibility of Mismatch Repair Testing of Colorectal Cancer Patients in a Low-Middle-Income Country

# Workflow for Standardization of CRC histopathological services

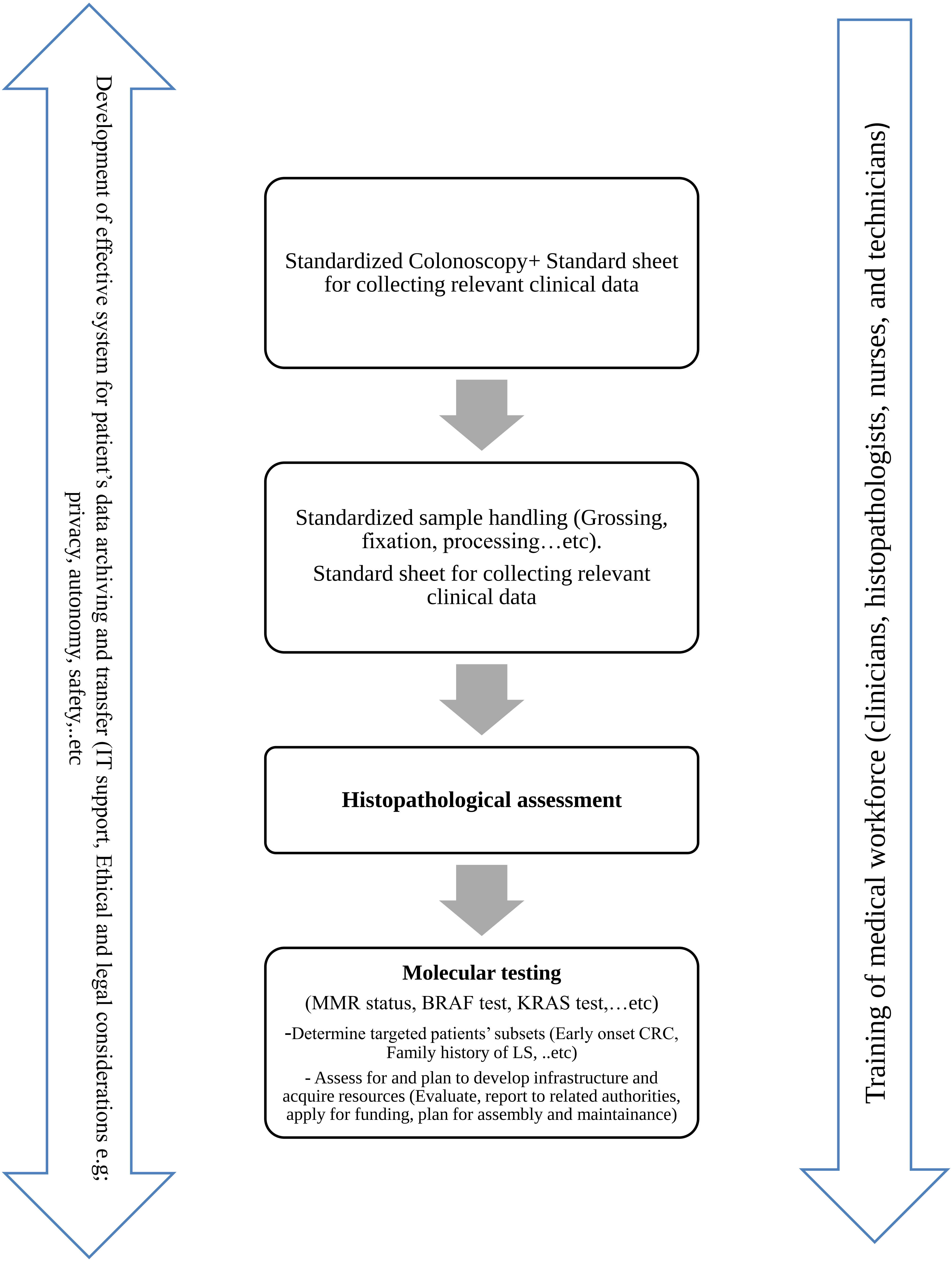

Supplement: Supplementary file 1 — Supplementary Information. [file 41598_2022_14644_MOESM1_ESM.pdf]
